# Pleural empyema by *Parvimonsd micra* in an immunocompetent patient: a case report

**DOI:** 10.17843/rpmesp.2023.401.11956

**Published:** 2023-03-20

**Authors:** Stalin Vilcarromero, Max Small, Alexis Lizarzaburu, Abel Rivadeneyra-Rodriguez

**Affiliations:** 1 Infectious Diseases Service of the Hospital Edgardo Rebagliati Martins, EsSalud, Lima, Peru. nfectious Diseases Service of the Hospital Edgardo Rebagliati Martins EsSalud Lima Peru; 2 Pneumology Service of the Hospital Edgardo Rebagliati Martins , EsSalud, Lima, Peru. Servicio de Neumología del Hospital Edgardo Rebagliati Martins EsSalud Lima Peru; 3 Odontostomatology Service of the Hospital Edgardo Rebagliati Martins, EsSalud, Lima, Peru. Odontostomatology Service of the Hospital Edgardo Rebagliati Martins EsSalud Lima Peru

**Keywords:** Pleural Empyema, Treatment, Gram-Positive Bacteria, Anaerobic Bacteria, Pericoronitis, MALDI-MS

## Abstract

We present the case of a young immunocompetent patient, with a history of pulmonary tuberculosis, who attended the hospital with a subacute clinical picture of persistent fever, weight loss, dyspnea and abolition of vesicular murmur. Chest CT scan showed an extensive empyema in the left hemithorax. Samples were taken for detection of common germs. Then, a chest drainage tube was placed and antibiotic therapy started. The MALDI-TOF MS test identified Parvimonas micra, an anaerobic bacterium, commensal to the oral flora, associated with severe periodontitis, but rarely reported in cases of pleural empyema, especially in immunocompetent patients. Gingivitis and pericoronaritis of the third molar were diagnosed during oral evaluation. The patient progressed favorably. Parvimonas micra should be considered as a possible etiological agent in cases of subacute or chronic pleural empyema, in addition to mycobacteria. Tests such as MALDI-TOF MS or 16S rRNA sequencing, chest tube placement, empirical antibiotic coverage and an adequate oral evaluation should be considered in these cases.

## INTRODUCTION

*Parvimonas micra (P. micra*) is a gram-positive anaerobic oral commensal bacterium, frequently associated with periapical lesions, chronic periodontitis, peri-implantitis and, occasionally, severe polymicrobial infections. *P. micra* is difficult to culture and identify ^1^, but it has been recognized as an emerging pathogen in patients with comorbidities or immunosuppression by the use of new microbiological diagnostic methods such as MALDI-TOF MS mass spectrometry and 16S rRNA sequencing. 

Pulmonary infections by *P. micra*, although rare, include pulmonary abscess, pleural effusion and empyema, which have not been previously reported in Peru.

We present the case of a young patient, without acquired immunodeficiency, with inadequate dental hygiene and pericoronitis of the third molar, who presented an infection by *P. micra,* which caused a large pleural empyema that responded adequately to drainage by thoracotomy tube and intravenous antibiotic treatment. We discuss clinical aspects and antibiotic management.

## CASE REPORT

We present the case of a 28-year-old male patient, from a peri-urban area of Lima, who worked as a motorcycle taxi driver. He had a history of complete individualized treatment, five years ago, for pulmonary tuberculosis resistant to isoniazid, streptomycin and ethionamide. The patient attended the emergency department of the Edgardo Rebagliati Martins National Hospital with a disease duration of 34 days presenting persistent fever, chills, weight loss, dyspnea and leukocytosis.

**Figure 1:**
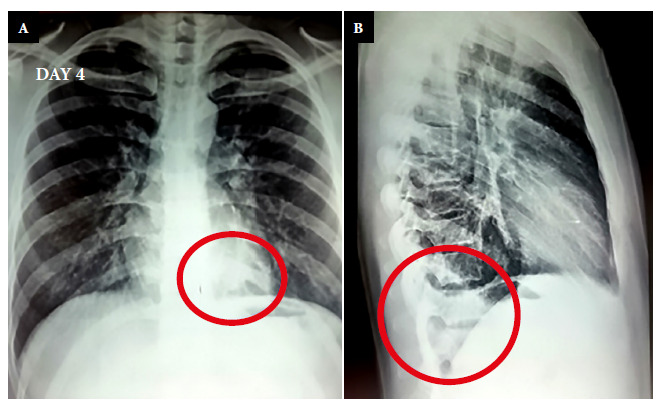
Posteroanterior and lateral chest radiographs on day 4 of the disease. (A) An opacity can be found in the anteroposterior face at the left paravertebral level (circle). (B) An opacity can be found in the lateral face at the level of the last dorsal vertebrae that blocks the costodiaphragmatic angle on that side (circle).

Initially, the patient presented moderate left rib cage pain, palpitations and high fever, and was treated by a private practice physician. The electrocardiogram showed no alterations and the chest X-ray on day 4 of illness (day 4) was reported as “normal” (Figure 1), but the intense fever (38°C), chills and leukocytosis with neutrophilia (Table 1) persisted. In addition, the patient lost weight and presented abdominal pain. Serology for immunodeficiency virus infection (HIV 1-2) and smear microscopy (BK) for Koch’s bacillus in three sputum samples were nonreactive and negative, respectively. Abdominal ultrasound and stool tests showed no alterations. The patient was hospitalized due to the persistence of these symptoms.

**Table 1:** Laboratory tests according to time of illness and hospitalization.

Parameters	Units	Normal values	DI 6	DI 15	DI 34	DI 38	DI 40	DI 42	DI 46	DI 50
					DH 1	DH 5	DH 7	DH 9 ^a^	DH 13	DH 17
Leucocytes	u/mm^3^	4160-10,570	14,380	14,440	20,690	28,450	-	19,510	9680	-
Stab cells	%	-	0	0	0	0	-	11	0	-
Neutrophiles	u/uL	1500-7500	12,654	11,150	16,600	23,980	-	16,400	7070	-
Lymphocytes	u/uL	1000-5200	719	1760	2080	2550	-	1420	1460	-
Platelets	u/mm^3^	150,000-450,000	280,000	573,000	573,000	562,000	-	519,000	589,000	-
Hemoglobin	g/dL	14-16	14.8	13.9		11.0	-	-	-	-
C reactive protein (PCR)	mg/dL	0-1	Negative	-	22.79	25.64	-	27.17	9.4	4.1
Creatinine	mg/dL	0.5-1.3	1.25	-	0.97	0.94	-	0.8	0.8	-
Urea	mg/dL	15-39	46	-	26	22.89	-		22.89	-
Glucose	mg/dL	70-105	118	-	122	75	-	70	73	-
Fibrinogen	mg/dL	200-400	-	-	-	670	637	-	-	-
Albumin	g/dL	3.2-4.8	-	-	3.86	2.79	-	2.9	3.2	-

DI: day of illness, DH: day of hospitalization.

a Day on which thoracocentesis was performed

### Clinical findings

Physical examination showed that the patient was in fair condition, thin, with a heart rate of 110 bpm, blood pressure of 100/70 mmHg, oxygen saturation of 96% without breathing support, moderate dehydration, and abolition of vesicular murmur in the middle and lower third of the left hemithorax.

### Schedule

Chills and fever persisted during hospitalization; leukocytes and C-reactive protein (CRP) also increased sharply (Table 1). Thoracentesis was performed on day 9 of hospitalization (DH 9)/day 42 of illness (DI 42) and a thoracic drainage tube was placed (TDT) in the left hemithorax. Samples for analysis were obtained from the 600 ml of purulent and greenish secretion that were drained initially. In DH 10 (DI 43) the patient started receiving intravenous antibiotic coverage.

**Figure 2:**
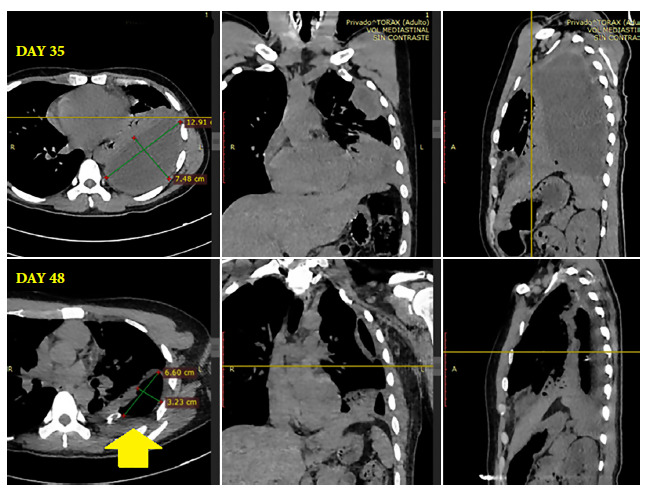
Computed tomography images. Axial view with sagittal and coronal reconstruction, on day 35 of illness shows a liquid collection of homogeneous content, with axial diameters of 12.9 x 7.5 cm and surrounded by a thick wall located, for the most part, in the posterior region. The image on day 48 of hospitalization shows an asymmetric, thick-walled cavity, with axial diameters of 6.6 cm x 3.2 cm, with a hydro-aerial level (yellow arrow), with decreased empyema volume, and adjacent pleural thickening.

At DH 12 (DI 45), the patient no longer had fever or leukocytosis, and neutrophilia and CRP had decreased (Table 1). The discharge was no longer purulent. CT scan at DH 15 (DI 48) showed a decrease in empyema volume by more than 50% (Figure 2) and chest ultrasound at DH 22 (DI 55) quantified an empyema volume of just 73 ml.

### Diagnostic evaluation

Initial chest CT scan showed extensive pleural empyema in the left hemithorax (Figure 2). The patient was diagnosed as an immunosuppressed and probable case of tuberculosis, and underwent serology tests for HIV 1-2 and human T-cell lymphotropic virus type 1 and 2 (HTLV 1-2), which were non-reactive. Culture and smear tests for *Mycobacterium tuberculosis* in sputum and urine were also negative.

The empyema samples, obtained by thoracentesis, were processed for the detection of *Mycobacterium tuberculosis* and common germs. For the former, the MGIT (Mycobacterium Growth Indicator Tube) automated liquid medium system and the GeneXpert MTB/RIF automated real-time PCR test were used, both of which were negative. The MALDI-TOF MS test was used for detecting common germs and was positive for *P. micra*. In this case it was not possible to perform an antibiogram.

The MALDI (Matrix-Assisted Laser Desorption/Ionization) technique, coupled to a TOF (time of flight) analyzer, is an ionization technique used in mass spectrometry, which allows the analysis of ribosomal proteins, typically between 2000 to 20 000 Da, from previously isolated bacteria ^2^, and compares them with ribosomal proteins from other bacteria in its database. In this way, aerobic and anaerobic pathogens ^3^ are identified quickly and reliably.

### Therapeutic intervention

Patient management included empyema sampling for etiologic diagnosis, placement of TDT, initiation of antibiotic coverage and dental evaluation. Empirical treatment started prior to the identification of *P. micra* with piperacillin/tazobactam, at a dose of 4.5 grams intravenous every 6 hours. The patient showed clinical, imaging and laboratory improvement after two weeks, therefore the TDT was removed. The same intravenous antibiotic treatment continued in an associated clinic for six weeks, then the possibility of decortication was evaluated.

Scarce subgingival calcified plaque, mostly in the posterior teeth and partially erupted congestive gingiva at the level of the lower third molars (teeth 38 and 48) were found during dental evaluation, which is compatible with chronic pericoronitis. Face tomography showed a chronic infectious process and the retention of teeth 38 and 48, which would require, later on, defocalization by means of complex exodontia and integral odontological treatment.

## DISCUSSION

To our knowledge, this is the first case of pleural empyema associated with *P. micra* reported in Peru. Other reported cases also present subacute or chronic disease with a prehospital time ranging from two weeks to several months ^4^^,^^5^. This would allow sufficient time to develop pleural thickening and also a possible delay in diagnosis. The patient sought medical attention with an illness time of four days, but the scarce pulmonary symptomatology together with an inadequate radiological interpretation, prevented early diagnosis. On admission to the hospital, the subacute symptoms of fever, chills and low weight, together with the history of previous tuberculosis, suggested that tuberculosis and HIV infection should be ruled out, further delaying the diagnosis. Another important aspect was the large volume of empyema at the time of drainage, which occupied more than 50% of the left hemothorax, with an initial drainage of 600 ml; other reports describe drainages of up to 1500 ml ^4^^,^^5^.

*P. micra* is a gram-positive, strictly anaerobic coccus that is part of the oral commensal flora, and has been frequently isolated in people with severe periodontitis ^6^. Poor dental hygiene is a determinant that favors an increased flora of *P. micra*. The patient did not have periodontitis, but had inadequate dental hygiene, chronic gingivitis and pericoronitis caused by partial eruption of teeth 38 and 48 (commonly known as “wisdom teeth”). The high prevalence of this bacterium has been previously reported, it has also been found to be a predictor of third molar pericoronitis ^7^. This may have contributed to increased local inflammation and thus bacterial spread to other tissues, such as the lung, causing empyema. Affections such as brain abscess, liver abscess, spondylodiscitis, skin and soft tissue infections have been reported ^8^^-^^11^, being vertebral involvement the most frequent ^12^.

Many cases of spondylodiscitis, endocarditis, and bacteriemia have been reported on elderly patients, cancer patients or in patients after surgery, which suggests that either immunodeficiency or postoperative stress could be risk factors ^8^. The patient, a young adult, did not present acquired infectious immunodeficiency due to HIV or HTLV 1-2, nor did he have comorbidities or used immunosuppressive medication, but he did show moderate hypoalbuminemia during hospitalization, which improved with diet and the treatment.

In the thorax, lung abscesses, empyema, and wall abscesses associated with *P. micra* infection have been reported, although infrequently ^12^. The advent of new microbiological diagnostic methods such as MALDI-TOF mass spectrometry have allowed greater detection of anaerobic bacteria such as *P. micra*, which is considered an emerging pathogen.

Although there is no clinical guideline for the management of empyema due to *P. micra*, antimicrobial susceptibility data from case reports can be used to guide therapeutic management. Treatment should include empyema drainage and appropriate antibiotic management. Since *P. micra* is an anaerobe of the oral cavity, it is theoretically possible to use penicillin, clindamycin, metronidazole, amoxicillin/clavulanic acid. In the case of empyema caused by *P. micra* and *Aggregatibacter aphrophilus*, Rodriguez-Segade *et al*. reported that placing a TDT with fibrinolytic and initially using imipenem and linezolid to then de-escalate to amoxicillin/clavulanic acid for four weeks showed successful results ^5^.

Potter *et al*. isolated *P. micra* sensitive to amoxicillin/clavulanic acid, which was treated by placing a TDT, pleural irrigation and use of fibrinolytic with intravenous coverage of amoxicillin/clavulanic acid (1g/125mg) three times a day plus ofloxacin 300 mg twice a day for 16 days, with favorable recovery of the patient ^4^. Cobo *et al*. reported the case of a patient with fever, leukocytosis, elevated CRP and identification of the pathogen in the pleural fluid; they used the E-test method of susceptibility to penicillin, amoxicillin/clavulanic acid, piperacillin/tazobactam, clindamycin, metronidazole, and imipenem with good response to a ten-day treatment with piperacillin/tazobactam and daptomycin ^12^. Yun *et al*. reported the case of a large volume lung abscess found by CT scan, where *Actinomyces odontolyticus* and *P. micra* were found after the abscess was drained with a pigtail catheter; the latter was sensitive to penicillin, amoxicillin, clindamycin, however, treatment started with intravenous ampicillin/sulbactam and then with amoxicillin/clavulanic acid for six months with complete resolution of the abscess ^13^.

A report on the *in vitro* antimicrobial resistance of isolates obtained from the subgingival flora of patients with severe periodontitis from private facilities in the United States found that resistance was more frequent in 2016, compared to 2006, for doxycycline (11.3% vs. 0.3%) and clindamycin (47.3% vs. 2.0%), while resistance to amoxicillin (2.3% vs. 1.0%) and metronidazole (0% vs. 0.3%) remained low; therefore, the authors recommended caution when using doxycycline and clindamycin ^6^. In the present case, due to the lack of an antibiogram, the prolonged hospitalization and good clinical evolution, the intravenous coverage continued for four weeks and then it changed to oral therapy, probably amoxicillin/clavulanic acid, which has shown to be sensitive according to reported cases *P. micra* isolated in pleural empyema and other presentations ^12^.

Two reported cases of pleural empyema due to *P. micra*^4^^,^^5^ presented caries and poor dental hygiene, while the case of pleural effusion did not report periodontitis or gingivitis, but suggested that the orotracheal tube was a possible entry into the lung; periodontitis was described in the case of pulmonary abscess. In the review of *P. micra* infection cases by Cobos *et al*, 16 (76%) of the patients had a history of a past dental procedure (extraction) due to periodontitis, apical abscess or dental caries ^12^. In the present case, the patient was diagnosed with gingivitis plus chronic pericoronitis caused by partial eruption of teeth, which would require exodontia in second instance to prevent future complications.

A limitation of this report is the fact that an antibiogram was not performed. It should also be noted that, although oral pathology was found, it cannot be confirmed that the lesions had a higher concentration of this bacterium and that, from there, it spread to the pleura.

In conclusion, dental evaluation should be considered in all immunosuppressed or immunocompetent patients with subacute or chronic empyema and *Parvimonas micra* infection should be included in the differential diagnosis, in addition to tuberculosis. Management should include a chest drainage tube, sampling for anaerobic cultures, identification by MALDI-TOF MS mass spectrometry or 16S rRNA sequencing plus antibiogram, as well as initial empirical treatment according to the local microbiological map or with metronidazole or amoxicillin/clavulanic acid as options.
