# Supplementary material for: Pleural empyema by Parvimonsd micra in an immunocompetent patient: a case report
Source: Rev Peru Med Exp Salud Publica. 2023 Mar 20;40(1):99–104. doi: 10.17843/rpmesp.2023.401.11956 (PMC10953646; doi:10.17843/rpmesp.2023.401.11956)
Supplement: Translation

# Translation: Empiema pleural por Parvimonas micra en un paciente inmunocompetente: reporte de un caso

**Authors:** Stalin Vilcarromero, Max Small, Alexis Lizarzaburu, Abel Rivadeneyra-Rodriguez

**Affiliations:** 1 Servicio de Infectología del Hospital Edgardo Rebagliati Martins, EsSalud, Lima, Perú.; 2 Servicio de Neumología del Hospital Edgardo Rebagliati Martins, EsSalud, Lima, Perú.; 3 Servicio de Odontoestomatologia del Hospital Edgardo Rebagliati Martins, EsSalud, Lima, Perú

**Keywords:** Empiema Pleural, Tratamiento, Bacterias Grampositivas, Bacterias Anaerobias, Pericoronitis, MALDI-MS

## Abstract

Se presenta el caso de un paciente joven inmunocompetente, con antecedente de tuberculosis pulmonar, que acude al hospital por un cuadro clínico subagudo de fiebre persistente, baja de peso, disnea y abolición del murmullo vesicular. La tomografía de tórax mostró un extenso empiema en hemitórax izquierdo. Se le toman muestras para detección de gérmenes comunes y se le colocan un tubo de drenaje torácico y se inicia antibioticoterapia. La prueba de MALDI-TOF MS identificó a *Parvimonas micra,* una bacteria anaerobia, comensal de la flora oral, asociado a periodontitis severa, escasamente reportado en empiema pleural, especialmente, en personas inmunocompetentes. En la evaluación odontológica se realizó el diagnóstico de gingivitis y pericoronaritis de la tercera molar. El paciente evolucion*ó* favorablemente. Se sugiere que, en casos de empiemas pleurales subagudos o crónicos, se debe considerar, además de las micobacterias, como agente etiológico al *Parvimonas micra*, y optar por exámenes como MALDI-TOF MS o secuenciamiento del 16S rRNA, colocación de tubo de tórax, cobertura antibiótica empírica y evaluación odontológica.

## INTRODUCCIÓN

*Parvimonas micra* (*P. micra*) es una bacteria anaerobia grampositiva, comensal de la microflora oral humana, frecuentemente asociado a lesiones periapicales, periodontitis crónica, periimplantitis y, ocasionalmente, a infecciones polimicrobianas graves. Es de difícil cultivo e identificación ^1^, pero, el uso de nuevos métodos diagnósticos microbiológicos tales como la espectrometría de masas MALDI-TOF MS y el secuenciamiento del 16S rRNA está permitiendo reconocerlo como un patógeno emergente en pacientes con comorbilidades o inmunosupresión.

Las infecciones pulmonares descritas por esta bacteria, aunque raras, incluyen absceso pulmonar, efusión pleural y empiema, los cuales no han sido reportados previamente en el Perú.

Se presenta el caso de un paciente joven, sin inmunodeficiencia adquirida, con inadecuada higiene dental, con pericoronaritis de la tercera molar, que presenta una infección por *P. micra* que ocasiona un empiema pleural de gran volumen y que responde adecuadamente al drenaje mediante tubo de toracotomía y tratamiento antibiótico endovenoso. Se discuten aspectos clínicos y del manejo antibiótico.

## REPORTE DE CASO

Paciente de 28 años, de sexo masculino, residente en un área periurbana de Lima, que labora como mototaxista. Tiene antecedente de tratamiento individualizado completo, hace cinco años, por tuberculosis pulmonar resistente a isoniazida, estreptomicina y etionamida. Acude al servicio de emergencia del Hospital Nacional Edgardo Rebagliati Martins con un tiempo de enfermedad de 34 días caracterizado por fiebre persistente, escalofríos, baja de peso, disnea y leucocitosis.

Inicialmente, el paciente fue atendido por un médico particular, con un cuadro clínico de dolor en la parrilla costal izquierda, de moderada intensidad, junto a palpitaciones y fiebre de alta intensidad. El electrocardiograma no mostró alteraciones y la radiografía de tórax del día 4 de enfermedad (día 4), fue reportada como «normal» (Figura 1), pero la fiebre intensa (38 °C), los escalofríos y la leucocitosis con neutrofilia (tabla 1) persistieron. Además, el paciente presentó baja de peso y dolor abdominal. Se le solicitó serología para la infección por el virus de inmunodeficiencia (VIH 1-2) y baciloscopia (BK) para la detección del bacilo de Koch, en tres muestras de esputo, que resultaron no reactivos y negativos, respectivamente. La ecografía abdominal y los exámenes de heces no mostraron alteraciones. Por persistencia de estos síntomas, el paciente fue hospitalizado.

**Figura 1:** Las radiografias posteroanteriores y lateral de tórax del día 4 de enfermedad. (A) En la incidencia anteroposterior a nivel paraverte-bral izquierdo se aprecia una opacidad (círculo). (B) En la incidencia lateral se observa una opacidad a nivel de la ultimas vertebras dorsales que obtura el angulo costodiafragmatico de ese lado (círculo).

**Tabla 1:** Exámenes de laboratorio según tiempo de la enfermedad y de hospitalización.

Parámetros	Unidades	Valores normales	DE 6	DE 15	DE 34	DE 38	DE 40	DE 42	DE 46	DE 50
					DH 1	DH 5	DH 7	DH 9 ^a^	DH 13	DH 17
Leucocitos	u/mm^3^	4160-10 570	14 380	14 440	20 690	28 450	-	19 510	9680	-
Abastonados	%	-	0	0	0	0	-	11	0	-
Neutrófilos	u/uL	1500-7500	12 654	11 150	16 600	23 980	-	16 400	7070	-
Linfocitos	u/uL	1000-5200	719	1760	2080	2550	-	1420	1460	-
Plaquetas	u/mm^3^	150 000-450 000	280 000	573 000	573 000	562 000	-	519 000	589 000	-
Hemoglobina	g/dL	14-16	14,8	13,9		11,0	-	-	-	-
Proteína C reactiva (PCR)	mg/dL	0-1	Negativo	-	22,79	25,64	-	27,17	9,4	4,1
Creatinina	mg/dL	0,5-1,3	1,25	-	0,97	0,94	-	0,8	0,8	-
Urea	mg/dL	15-39	46	-	26	22,89	-		22,89	-
Glucosa	mg/dL	70-105	118	-	122	75	-	70	73	-
Fibrinógeno	mg/dL	200-400	-	-	-	670	637	-	-	-
Albumina	g/dL	3,2-4,8	-	-	3,86	2,79	-	2,9	3,2	-

DE: día de enfermedad, DH: día de hospitalización

a Dia en el que se realizó la toracocentesis

### Hallazgos clínicos

En el examen físico, el paciente se encontró en regular estado general, adelgazado, con frecuencia cardiaca de 110 latidos por minuto, presión arterial de 100/70 mmHg, saturación de oxígeno de 96% sin apoyo oxigenatorio, moderada deshidratación y abolición del murmullo vesicular en tercio medio e inferior del hemitórax izquierdo.

### Calendario

Durante la hospitalización, el paciente persistió con fiebre, escalofríos y marcado incremento de los leucocitos y de la proteína C reactiva (PCR) (tabla 1). El día 9 de hospitalización (DH 9) o día 42 de enfermedad (DE 42), se le realizó toracocentesis más colocación de un tubo de drenaje torácico (TDT) en el hemitórax izquierdo. Se drena, inicialmente, 600 ml de secreción purulenta y verdosa, del cual se obtienen muestras que son enviadas al laboratorio para su análisis. En el DH 10 (DE 43) le inician cobertura antibiótica por vía endovenosa.

En el DH 12 (DE 45), el paciente ya no tiene fiebre y, la leucocitosis, la neutrofilia y la PCR, han disminuido (tabla 1). La secreción ya no es purulenta. La tomografía del DH 15 (DE 48) mostró una disminución del volumen del empiema en más del 50% (figura 2) y la ecografía de tórax del DH 22 (DE 55) lo cuantificó, solamente, en 73 ml.

**Figura 2:** La tomografía computarizada, en corte axial con reconstrucción sagital y coronal, del día 35 de enfermedad evidencia una colección líquida de contenido homogéneo, con diametros axiales de 12,9 x 7,5 cm y rodeada de una gruesa pared localizada, en su mayor parte, en la región posterior. La tomografía del día 48 de hospitalización mostró una cavidad asimétrica, de paredes gruesas, con diametros axiales de 6,6 cm x 3,2 cm, con un nivel hidroaéreo (flecha amarilla), con disminución del volumen del empiema, y engrosamiento pleural adyacente.

### Evaluación diagnóstica

La tomografía de tórax inicial mostró la presencia de un empiema pleural extenso en hemitórax izquierdo (figura 2). Se consideró, como un caso probable de tuberculosis en un paciente inmunosuprimido y se le realizaron nuevos exámenes de serología para VIH 1-2 y para el virus linfotrópico humano de células T tipo 1 y 2 o HTLV 1-2, los cuales resultaron no reactivos. Las pruebas de cultivo y baciloscopia para *Mycobacterium tuberculosis* en esputo y orina, también fueron negativos.

Las muestras del empiema, obtenidas mediante toracocentesis, fueron procesadas para la detección de *Mycobacterium tuberculosis* e identificación de gérmenes comunes*.* Para el primero, se usó el sistema automatizado en medio líquido MGIT (*Mycobacterium Growth Indicator Tube*) y la prueba de PCR en tiempo real automatizada GeneXpert MTB/RIF que resultaron negativos. Para el segundo, se utilizó la prueba de MALDI-TOF MS que resulto positivo a *P. micra*. En este caso no fue posible realizar un antibiograma.

La técnica MALDI (*Matrix-Assisted Laser Desorption/Ionization*), acoplada a un analizador TOF (*time of flight*), es una técnica de ionización usada en la espectrometría de masa, que permite el análisis de las proteínas ribosomales, típicamente entre 2000 a 20 000 Da, de las bacterias aisladas previamente ^2^, y las compara con las proteínas ribosomales de otras bacterias que tiene en su base de datos. De este modo, se logra la identificación de los patógenos aerobios y anaerobios ^3^, de forma rápida y confiable.

### Intervención terapéutica

Para el manejo del paciente, se consideró la toma de muestras del empiema para el diagnóstico etiológico, colocación del TDT, inicio de cobertura antibiótica y evaluación odontológica. Previo a la identificación de *P. micra,* se inició tratamiento empírico con piperacilina/tazobactam, a dosis de 4,5 gramos endovenoso cada 6 horas. Luego de dos semanas, al observar notable mejoría clínica, imagenológica y laboratorial, se decide el retiro del TDT y la continuación del mismo tratamiento antibiótico endovenoso en una clínica asociada, hasta completar seis semanas, para luego, evaluar la posibilidad de decorticación.

En la evaluación odontológica se encontró escasa placa calcificada subgingival, a predominio de las piezas dentales del sector posterior, encía congestiva, a nivel de los terceros molares inferiores (pieza 38 y 48), parcialmente erupcionados, lo cual es compatible con pericoronaritis crónica. El estudio tomográfico de macizo facial evidenció el proceso infeccioso crónico y la retención de las piezas 38 y 48, lo cual va a requerir, posteriormente, desfocalización mediante exodoncias complejas y tratamiento odontológico integral.

## DISCUSIÓN

Hasta donde se conoce, este es el primer caso de empiema pleural asociado a *P. micra* que se reporta en el Perú. Como se ha observado en otros casos, el cuadro es usualmente subagudo o crónico con un periodo prehospitalario entre dos semanas a varios meses ^4^^,^^5^. Esto permitiría, un tiempo suficiente para desarrollar engrosamiento pleural y también un posible retraso en el diagnóstico. El paciente acudió por atención médica con un tiempo de enfermedad de cuatro días, pero la escasa sintomatología pulmonar junto a una inadecuada interpretación radiológica, alejaron la posibilidad de un diagnóstico temprano de la patología pulmonar. En su ingreso al hospital, el cuadro subagudo, de fiebre, escalofríos y baja de peso sumado al antecedente de haber tenido tuberculosis, sugería insistir en el descarte de tuberculosis e infección por VIH, lo que retraso aún más el diagnóstico. Otro aspecto importante fue el gran volumen del empiema en el momento del drenaje, que ocupaba más del 50% de hemotórax izquierdo, con un drenaje inicial de 600 ml, mientras que en otros reportes se describen drenajes de hasta 1500 ml ^4^^,^^5^.

Se conoce que *P. micra* es un coco grampositivo, anaeróbico estricto, que forma parte de la flora comensal oral, y ha sido frecuentemente aislado en personas con periodontitis severa ^6^. La mala higiene dental es un determinante que favorece una mayor flora de *P. micra*. El paciente no tenía periodontitis, pero sí una inadecuada higiene dental, gingivitis y pericoronaritis crónicas causado por erupción parcial de las piezas 38 y 48 (popularmente conocidos como «muelas del juicio»). Ya se ha reportado la gran prevalencia de esta bacteria y como predictor de pericoronaritis de la tercera molar ^7^. Esto puede haber contribuido a una mayor inflamación local y de ese modo, a una dispersión bacteriana hacia otros tejidos, como el pulmonar, causando empiema. Entre los otros órganos comprometidos se ha reportado absceso cerebral, absceso hepático, espondilodiscitis, infecciones de piel y tejidos blandos ^8^^-^^11^, siendo el vertebral el más frecuentemente comprometido ^12^.

Muchos de los casos de espondilodiscitis, endocarditis y de bacteriemia reportados han ocurrido en pacientes con edad avanzada, cáncer o posoperados, esto sugiere que tanto la inmunodeficiencia o el estrés posoperatorio podrían ser factores de riesgo ^8^. El paciente, adulto joven, no presentaba inmunodeficiencia infecciosa adquirida por VIH o HTLV 1-2, tampoco tenía comorbilidades o uso de medicación inmunosupresora, pero si mostró hipoalbuminemia moderada durante su hospitalización, lo que mejoro con la dieta y el manejo instaurado.

En tórax, se ha reportado, aunque infrecuentemente, absceso pulmonar, empiema y absceso de pared asociada a la infección por *P. micra*^12^. El advenimiento de nuevos métodos diagnósticos microbiológicos como la espectrometría de masas por MALDI-TOF ha permitido una mayor detección de las bacterias anaerobias como *P. micra,* al cual se le considera un patógeno emergente.

Aunque no existe una guía clínica para el manejo del empiema por *P. micra*, se puede considerar los datos de susceptibilidad antimicrobiana de los reportes de casos para guiar la conducta terapéutica. El tratamiento debe incluir drenaje del empiema y manejo antibiótico apropiado. Al tratarse de un anaerobio de la cavidad oral, teóricamente es posible tratarla con penicilina, clindamicina, metronidazol, amoxicilina/ácido clavulánico. En el caso de empiema por *P. micra* y *Aggregatibacter aphrophilus*, reportado por Rodríguez-Segade *et al*, se colocó un TDT con fibrinolítico y se administró, inicialmente, imipenem y linezolid, para luego desescalar a amoxicilina/ácido clavulánico por cuatro semanas con total éxito ^5^.

En otro caso de empiema reportado por Potter *et al*, se aisló, únicamente, *P. micra* sensible a amoxicilina/ácido clavulánico, que fue manejado con colocación de un TDT, irrigación pleural y uso de fibrinolítico con cobertura endovenosa de amoxicilina/ácido clavulánico (1 g/125 mg) tres veces al día más ofloxacino 300 mg dos veces al día por 16 días, con recuperación favorable del paciente ^4^. Cobo *et al*, reportaron, un caso de un paciente con fiebre, leucocitosis, elevación de PCR y con identificación del patógeno en el líquido pleural, donde se usó el método de E-test de susceptibilidad a penicilina, amoxicilina/ácido clavulánico, piperacilina/tazobactam, clindamicina, metronidazol, e imipenem, y con buena respuesta al tratamiento por diez días con piperacilina/tazobactam y daptomicina ^12^. Yun *et al*, reportaron un caso de absceso pulmonar de gran volumen y con imagen tomográfica, que se drenó con catéter de pigtail, donde se aisló *Actinomyces odontolyticus* y *P. micra*, siendo este último sensible a penicilina, amoxicilina, clindamicina, sin embargo, fue tratado con ampicilina/sulbactam endovenoso y luego con amoxicilina/ácido clavulánico por seis meses con resolución completa del absceso ^13^.

La resistencia antimicrobiana observada «*in vitro*» en los aislamientos obtenidos de la flora subgingival en pacientes con periodontitis severa atendidos en establecimientos privados de Estados Unidos reportaron que la resistencia fue más frecuente el 2016, en comparación con el 2006, para la doxiciclina (11,3% *vs*. 0,3%) y la clindamicina (47,3% *vs.* 2,0%), mientras que la resistencia a la amoxicilina (2,3% *vs*. 1,0%) y al metronidazol (0% *vs.* 0,3%) se mantuvo baja por lo que recomiendan precaución con doxiciclina y clindamicina ^6^. En el presente caso, por carecer de antibiograma, estancia hospitalaria prolongada, y por la buena evolución clínica, se decidió completar la misma cobertura endovenosa por cuatro semanas y luego, optar por terapia vía oral, probablemente con amoxicilina/ácido clavulánico que mostraron tener sensibilidad según los casos reportados de *P. micra* aislados en empiema pleural y otras presentaciones ^12^.

Los dos casos de empiema pleural por *P. micra*^4^^,^^5^, presentaron caries y pobre higiene dental, mientras que el caso de efusión pleural no reportó periodontitis o gingivitis, pero sugería al tubo orotraqueal como factor de entrada al pulmón; y en el caso del absceso pulmonar, si se describió periodontitis. En la revisión de casos reportada por Cobos *et al*, de infección por *P. micra*, 16 (76%) de los pacientes presentaron antecedente de procedimiento dental (extracción) por periodontitis, absceso apical o caries dental ^12^^)^ . En el presente caso, el diagnóstico fue gingivitis más pericoronaritis crónica causada por erupción parcial de piezas dentarias, lo que requeriría de exodoncia en segunda instancia para prevenir complicaciones futuras.

Entre las limitaciones del presente reporte se debe mencionar que no se realizó un antibiograma, y aunque se encontró patología oral, no se puede afirmar que las lesiones tenían mayor concentración de esta bacteria y que, de allí, se dispersó a pleura.

En conclusión, en todo paciente inmunosuprimido o inmunocompetente, con cuadro de empiema, subagudo o crónico, debería considerarse la evaluación odontológica e incorporar, dentro del diagnóstico diferencial, además de tuberculosis, la infección por *Parvimonas micra*. El manejo debe incluir colocación de un tubo de drenaje torácico, toma de muestras para cultivos para anaerobios, identificación mediante espectrometría de masas MALDI-TOF MS o secuenciamiento del 16S rRNA más antibiograma, y tratamiento empírico inicial según mapa microbiológico local o con metronidazol o amoxicilina/ácido clavulánico como opciones, todavía, adecuadas en el presente.
